# The effects of 10-week plyometric training program on athletic performance in youth female handball players

**DOI:** 10.3389/fspor.2023.1193026

**Published:** 2023-07-14

**Authors:** Nawel Gaamouri, Mehrez Hammami, Yosser Cherni, Thomas Rosemann, Beat Knechtle, Mohamed Souhaiel Chelly, Roland van den Tillaar

**Affiliations:** ^1^ Research Laboratory (LR23JS01) “Sport Performance, Health and Society”, Higher Institute of Sport and Physical Education of Ksar Saîd, University of Manouba, Tunis, Tunisia; ^2^ Higher Institute of Sport and Physical Education of Ksar Said, University of Manouba, Tunis, Tunisia; ^3^ Institute of Primary Care, University of Zurich, Zurich, Switzerland; ^4^ Medbase St. Gallen Am Vadianplatz, St. Gallen, Switzerland; ^5^ Department of Sport Sciences and Physical Education, Nord University, Levanger, Norway

**Keywords:** stretch shortening cycle, peak power, 1-RM, upper limb, lower limb

## Abstract

**Purpose:**

The aim of the study was to investigate the effects of a 10-week plyometric training (PT) on changes of direction, jumping ability, repeated sprint ability, and both muscular strength and power in youth female handball players.

**Methods:**

Twenty-eight participants (age: 15.8 ± 0.2 years) were randomly divided into a plyometric group (PG; *n* = 14) or a control group (CG; *n* = 14). Significant (group × time) interaction was noted for change of direction (COD) [Modified agility *T*-test (T-half)], three jumping tests [squat jump (SJ), countermovement jump (CMJ) and standing long jump (SLJ)], repeated sprint ability (RSA), muscular strength (1-RM bench press and 1-RM half squat) and muscular power (force-velocity test for both upper and lower limb).

**Results:**

With a group × time interaction, the PG enhanced the T-half performance [*p* < 0.001, Δ = 10.4, *d* = 1.95 (large)] compared to the CG. The PG enhanced the jump performance over SJ [*p* = 0.009, Δ = 18.3, *d* = 0.72 (medium)], CMJ [*p* = 0.005, Δ = 20.7, *d* = 0.79 (medium)] and SLJ [*p* < 0.001, Δ = 24.5, *d* = 2.25 (large)]. Three of four RSA scores increased significantly in the PG compared to the CG [*p* < 0.001, Δ = 2.76, *d* = 1.11 (large); *p* < 0.001, Δ = 2.72, *d* = 1.23 (large); and *p* < 0.001, Δ = 2.75, *d* = 1.21 (large), in best time (RSA-BT), mean time (RSA-MT) and total time (RSA-TT), respectively]. In contrast, group × time interactions revealed no significant differences in both 1-RM bench press and 1-RM half squat performance between PG and CG. Regarding the force velocity performance, the PG enhanced 3 of 4 force velocity scores for the upper limb performance [*p* < 0.001, *d* = 1 (large); *p* < 0.001, *d* = 1.13 (large) and *p* = 0.012, *d* = 0.72 (medium) for the peak power in these two forms (W and W·kg-1), and maximal pedalling velocity, respectively], and 2 of 4 force velocity scores for the lower limb performance [*p* = 0.045, *d* = 0.56 (medium); and *p* = 0.021, *d* = 0.65 (medium) for the peak power in these two forms (W and W·kg-1), respectively].

**Conclusion:**

It was concluded that additional PT performed two times per week during 10 weeks enhances measures related to game performance, such as COD, jump ability, RSA, and power in youth female handball players.

## Introduction

Handball is a team sport that requires the use of several key parameters of performance (e.g., anthropometric, physiological, psychological, and motor skill characteristics) (1–3). The physical and physiological characteristics and the on-court performances of handball players have recently been reviewed (4, 5). It is a contact sport, which includes jumping, running, changes of direction and arm throwing ball as prominent features of performance (6–9). Muscle strength and power are key components of fitness performance, required in many explosive actions (e.g., jump, change of direction, throw, sprint) and constitute an essential part of any young athlete's overall training program (10). To maintain a high level of physical performance, both strength/power training should be carefully monitored throughout the competitive season.

PT programs could be very useful to develop lower/upper limb power for both male and female handball players (11–13). PT can play a significant role in the development of young female handball players. Plyometrics involves explosive movements that aim to enhance power, speed, and agility, which are essential attributes for handball players (14). Hence, handball coaches should perform specific PT to develop these physical qualities. This type of program (i.e., PT) consists of an eccentric muscle contraction followed by a concentric muscle contraction the aim of which is to improve the stretch-shortening cycle (i.e., time between eccentric contraction and eccentric contraction) and subsequently improve physical qualities (11, 13–17). PT is based on jumping (such as hurdle jump, drop jump, etc.), skipping and hopping exercises to benefit the shortening stretch cycle (18). Incorporating this type of exercise improves the ability of the muscle-tendon unit to produce maximum force in the shortest possible time (i.e., muscular strength and muscular power) (19), and subsequently the physical qualities. Leading world fitness and health organizations guidelines, review articles, and meta-analyses (14, 18, 20, 21) indicate that PT, if correctly done, can be very beneficial for adolescents.

The ability to closely mimic powerful actions essential for success in handball makes plyometric exercises an ideal method of resistance training activity for young female handball players. In previous studies, the effects of PT on young handball athletes (11, 13) were investigated. For instance, Hammami et al. (13) found increases in sprint speed, change of direction, jump ability and repeated change of direction after 10-week upper and lower PT in young female handball players aged 15.8 ± 0.2 years. Furthermore, Chaabene et al. (11) revealed that PT (2 weekly sessions for 8 weeks) improves measures of physical fitness (i.e., linear/change of direction speed, jumping, and RSA) in young female handball players aged 15.9 years old. In fact, Hammami et al. (12), found increases in both upper limb (handgrip force, back extensor strength, and medicine ball throwing) and lower limb [sprinting, change of direction (CoD), jumping] performance after 9 weeks of combined upper and lower limb PT in U14 female handball players. Controversy, Meszler and Vaczi (22), found no significant changes in T agility test scores, balance, hamstring strength or H:Q ratio after 7 weeks of PT in female basketball players aged younger than 17 years.

Recently, the review of Ramirez-Campillo et al. (23), found that scarce information is available in the literature on PT effects in female players (23). This implies that future studies including female participants are needed to provide more in-depth knowledge for coaches and practitioners. Moreover, a recently published systematic review outlined that PT studies on the effects on physical fitness in young female athletes suffer from numerous methodological shortcomings and are limited in number. A major limitation is the lack of controls which affects the veracity of findings (21). This implies that more controlled PT trials are needed in female players.

Therefore, the aim of this study was to determine how far the substitution of a short-term plyometric program for some existing drills within a regular in-season handball training program would enhance physical performance in young female handball players. A plyometric program was introduced into the normal in-season regimen for 10 weeks for participants, without increasing their total training time. Taking into consideration the previous investigations on this topic (11, 23), we expected that 10 weeks of PT would improve change-of-direction ability, jump height, repeated sprint ability, power and strength performance.

## Materials and methods

### Participants

The Gpower 3.0.10 program was used to calculate the minimal sample size needed in our study, with Z1-*β* = 1.03 (power = 85%) and Z/2 = 1.96 (alpha = 5%). The study of Meszler and Váczi (22) showed the mean ± SD of counter movement vertical jump as 33.52 ± 3.89 (cm) in the experimental group vs. 28.72 ± 6.66 (cm) in the control group, and considering a ratio of 1 control for every case, there was a need for a minimum of 11 experimental and 11 control subjects (24). In order to explore the effects of a short-term (i.e., 10 weeks) PT program on measures of athletic performance in youth female handball players, twenty eight youth female handball players from the same club were divided by playing position, and players from each position were then randomly assigned into a plyometric group (PG) (*n* = 14; age 15.7 ± 0.2 years; body mass 63.8 ± 3.3 kg; body height 1.65 ± 0.03 m; body fat 25.4 ± 4.1%; maturity-offset 2.9 ± 0.4 years) or a control group (CG) (*n* = 14; age 15.8 ± 0.2 years; body mass 63.3 ± 4.1 kg; body height 1.67 ± 0.03 m; body fat 24.6 ± 1.8%; maturity-offset 3.0 ± 0.4 years). They were examined by the team physician, with a particular focus on conditions that might preclude elastic band training, and all were found to be in good health (the player who is not in good health, excluded from the study). All participants were classified as highly trained athletes (25). They participated in national competitions for at least 5 years and they had 3 years’ experience of PT. All had already achieved a good overall physical preparation at the beginning of the season (a preliminary 6-week period of 6 training sessions per week). This preliminary phase was divided into 2 parts. The ﬁrst 3 weeks included a resistance training program which aimed to improve muscular endurance by light loads (30%–50% 1 repetition maximum). The second 3-week period was devoted to improving muscular power with higher loads (40%–60% 1 repetition maximum realized at high velocity), accompanied by friendly matches each weekend. The subjects continued to participate in 5 training sessions per week during September at the championship season. The experimental intervention of biweekly PT was undertaken during the second phase of the national championships (January to March). All participants had previously engaged in five to six training sessions per week (90–120 min each session). However, for 10 weeks, the EG replaced some of their handball-specific drills with a PT program, although the overall training volume remained comparable for the two groups. Any athlete missing more than 10% of the total training sessions and/or a player who is not in good health would be excluded from the study. During the intervention, the CG followed their usual handball training (i.e., mainly technical-tactical exercises, small-sided and simulated games, or injury prevention drills).

### Procedures

**Figure 1:**
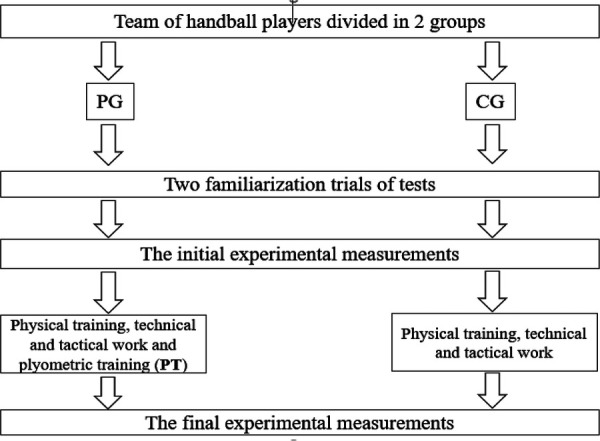
The diagram includes detailed information on the interventions received.

This current study examined whether 10-week of biweekly in-season PT would enhance certain performance-related capacities in initially well-trained youth female handball players relative to their peers who continued to follow their customary in-season training regimen [the CG followed their usual handball training (i.e., mainly technical-tactical exercises, small-sided and simulated games, or injury prevention drills)]. Two familiarization sessions were held, 2 weeks before baseline test session, to get participants acquainted with the tests. Measurements were made in a ﬁxed order over 4 days, immediately before and 4 days after the last plyometric training session the subjects were disallowed to participate in any exhausting exercise for 24 h before testing, and to consume any food or caffeine-containing drinks 2 h before testing. The training intervention was conducted during the in-season period of the year 2018–2019. Training and measurements were made at the same time of day (5:00–7:00 PM), under approximately the same environmental conditions (temperature: 20°C–25°C) on a wooden surface at the same time of day indoors handball hall. A standardized warm-up (10–20 min of low- to moderate-intensity aerobic exercise and dynamic stretching) preceded all tests. On the ﬁrst test day, participants made modified agility *T*-test (*T*-test), standing long jump (SLJ), and repeated sprint ability (RSA). The second day was devoted to upper limb force velocity test and 1-RM half squat. On the third day, anthropometric measurements were followed by jumping ability [i.e., squat jump (SJ), counter-movement jump (CMJ)]. On the fourth and last day, 1-RM bench press and lower limb force velocity test were completed. All tests were scheduled at least 48 h after the most recent training session or competition and under the same experimental conditions. Participants were instructed to use the same athletic shoes and clothes during the pre- and post-testing (Figure 1).

### Testing procedures

#### Anthropometry

Anthropometric measurements included standing and sitting body height (stadiometer accuracy of 0.1 cm; Holtain, Crosswell, Crymych, Pembs, United Kingdom) and body mass (0.1 kg; Tanita BF683W scales, Munich, Germany). The overall percentage of body fat was estimated from the biceps, triceps, subscapular, and suprailiac skinfolds, using the equations of Durnin and Womersly (26) for children and adolescent females:

%Bodyfat=(495/D)−450

where *D* = 1.1369–0.0598 (Log sum of 4 skinfolds)

Maturity offset status was calculated from peak height velocity ^23^:

Maturity offset = −9.38 + (0.000188 × leg length × sitting height)+ (0.0022 × age × leg length) + (0.00584 × age × sitting height) + (0.0769 × weight/height ratio):

#### Modified agility *T*-test

As previously described (27) the modified *T*-test was performed to determine speed with directional changes such as forward sprinting, left and right shuffling, and backpedalling. Performance times were recorded to the nearest 0.01 s by paired single beam photocells (Microgate, Bolzano, Italy). Each player performed two attempts with 5 min of rest between them, and the best attempt was used for analyses.

#### Vertical jumps

Jump height was evaluated using an infrared photocell mat related to a digital computer (Optojump System, Microgate SARL, Bolozano, Italy). Flight times were measured with a precision of 1/1,000 s, allowing calculation of jump heights. Players started the SJ at a knee angle of 90 degrees, and made a vertical jump by pushing upwards, keeping their legs straight (28). The CMJ began from an upright position, subjects making a rapid downward movement to a knee angle of 90 degrees and simultaneously beginning to push-off. One minute of rest was permitted between the three trials of each test, the highest jump being used in subsequent analyses. Each player performed two attempts with 5 min between them, and the best attempt was used for analyses.

#### Standing long jump

The starting position required subjects to stand with their feet at shoulders’ width behind a line marked on the ground and their arms in neutral position (28). On the command ready, set, go, participants executed a countermovement with their legs and arms and jumped at maximal effort in the horizontal direction. Participants had to land with both feet at the same time and were not allowed to fall forward or backward. The horizontal distance between the starting line and the heel of the rear foot was evaluated via tape measure to the nearest 1 cm. Each player performed two attempts with 5 min between them, and the best attempt was used for analyses.

#### Repeated sprint ability (RSA) test

After a standardized warm-up, the shuttle RSA test involved 6 repetitions of 2 m × 20 m shuttle sprints (approximately 7 s running time). In this test, sprints were repeated every 20 s (29–31). An active recovery was allowed through a quick walk back to the starting line. Three seconds before starting each sprint, players took an individually chosen starting position 0.5 m behind the timing gate. A digital timer started automatically when the player passed the gate. Two timing gates (Microgate Srl; Race time 2. Light Radio, Bolzano, Italy) working in opposite directions allowed subjects to start the next run from the end where they had finished the preceding sprint. Strong verbal encouragement was provided throughout, and participants were asked to perform each sprint with maximal effort, avoiding pacing. Four scores were assessed: best sprint time (RSA-BT), mean sprint time (RSA-MT), total sprint time (RSA-TT) and fatigue index (RSA-FI), the last calculated as the percentage decrement: 100—(Total time/ideal time × 100); where the ideal time = 6 × RSA best time (31).

#### 1-RM half squat and bench press

Muscular strength of participants was evaluated by one maximum repetition (32), measured three times (just before starting the training program, at the fourth week and after 2 months). Thus, training loads (%RM) were accurately adjusted during the training program, following previous literature guidelines (24). First, the player was instructed to perform a light resistance warm-up from 10 to 12 repetitions in the assessed exercise. Then, a 1-minute rest was allowed. A warm-up load was added to allow the athlete to complete 3–5 repetitions (5%–10% for bench press and 10%–20% for leg press and back squat). A 2-minute rest time was provided. Again, a 5%–10% increase in the load was performed for bench press and 10%–20% for leg press and back squat. A 4-minute rest time was provided. The load was again increased for the athlete to attempt one maximum repetition. The load continued to be increased or decreased until the player completed one repetition with proper exercise technique. In both leg press and squat exercises, participants were asked to perform a thigh-knee 90° angle range of motion. In bench-press, also an arm-forearm 90° range of motion was defined as the final moment of the eccentric phase.

#### The force-velocity test

The lower limb force–velocity tests were executed on a standard cycle ergometer (model 894 E, Monark Exercise AB, Vansbro, Sweden). The corresponding maximal anaerobic power was calculated using the instantaneous peak velocity at each braking force. The maximal velocity (V0) was identified as the highest velocity attained without external loading. Peak power was defined as the power at which additional loading induced a decrease in power output. Parabolic relationships were determined only if we observed a decline of peak power over two successive braking forces.

Upper limb tests were made using an appropriately modified version of the same apparatus. Hand cranks replaced the pedals, and the saddle pillar was removed to avoid injuries. The ergometer was then mounted on a metal support that brought the crankshaft to shoulder level. The unrestrained subjects stood freely in front of the ergometer, with the exception that smaller participants were allowed to stand on a step.

The measured and calculated parameters for both tests contained Peak power of the upper (PP_UL_) and lower (PP_LL_) limbs, each expressed in Watts, W·kg^−1^ of total body mass, and the corresponding maximal forces (F0_UL_ and F0_LL_) and maximal velocities (V0_UL_ and V0_LL_). The force–velocity tests required short all-out sprints (duration about 7 s) using a suitable sequence of ergometer braking forces (33, 34). The force–velocity tests required short all-out sprints (duration about 7 s) using a suitable sequence of ergometer braking forces. After a 10-minute standardized warm-up, lower limbs tests began at a braking force equal to 2.5% of the participants’ body mass (33). After a 5-minute recovery, the braking was increased to 5%, 7.5%, 8.5%, 9.5%, 10.5%, and 11.5% of body mass in randomized order. The same sequence was performed again, until an additional load induced a decrease of power at each of 2 repetitions; this value was accepted as the PP. Six to 8 all-out sprints were generally performed in a session. The upper limbs protocol was similar, beginning with a braking force equal to 1.5% of the participants’ body mass. After a 10-minute warm-up, the braking was increased by 0.5% every bout, until the subject could not reach the previous peak of power in 2 successive bouts.

### Plyometric training program

The PT program completed a 10-week in-season with two training sessions per-week (Tuesday and Thursday), respectively, based on the players’ previous training records and research results (13, 23). PT drills were incorporated into their regular 90–120 min handball training routines, replacing some low-intensity technical-tactical handball exercises. Without counting competitive and friendly matches, the PT replacement activity represented <10% of the total training load. During the intervention, the CG followed their usual handball training (i.e., mainly technical-tactical exercises, small-sided and simulated games, and injury prevention drills).

The rating of perceived exertion RPE (35) was used to control the overall training load and ensure no differences between both groups. A standardized 8–12 min warm-up preceded each PT session, including low-intensity running, coordination exercises, dynamic movements (i.e., lunges, skips), sprints, and dynamic stretching for both upper and lower limb muscles. The intervention included push-up exercises for the upper limbs (both exercises performed at high velocity), and hurdling, lateral hurdling, and hurdle jumping (jumping with 180° rotation) exercises for the lower limbs. Exercises for the upper limbs were immediately followed by lower-limb exercises (i.e., 6–10 repetitions of dynamic push-ups + 6–8 repetitions of lower limb jumps), with no intervening rest periods (Table 1). The sequence of plyometric exercises for the upper and lower limbs lasted ∼10 s (20, 21, 23). A time of 30 s was fixed as a recovery time between sets. All plyometrics in general (i.e., upper and lower limb exercises) were performed with maximal effort, minimizing contact time in each repetition, and no resting was allowed between jumps.

**Table 1:** Plyometric training program.

	Weeks 1–2	Weeks 3–4	Weeks 5–6	Weeks 7–8	Weeks 9–10
	Set × Repetition	Set × Repetition	Set × Repetition	Set × Repetition	Set × Repetition
**Upper limb**					
Push-up	10 × 6	10 × 6	10 × 6	10 × 6	10 × 6
Contacts number	60	60	60	60	60
	Weeks 1–2	Weeks 3–4	Weeks 5–6	Weeks 7–8	Weeks 9–10
	H × S × R	H × S × R	H × S × R	H × S × R	H × S × R
**Lower limb**					
Hurdle jump	0.3 m × 2 × 6	0.3 m × 3 × 6	0.35 m × 2 × 6	0.35 m × 3 × 6	0.4 m × 2 × 6
Lateral hurdle jump	0.3 m × 2 × 6	0.3 m × 3 × 6	0.35 m × 2 × 6	0.35 m × 3 × 6	0.4 m × 2 × 6
Stretched leg jump	0.25 m × 2 × 6	0.25 m × 3 × 6	0.30 m × 2 × 6	0.30 m × 3 × 6	0.35 m × 2 × 6
Hurdle jump (jump with 180°)	0.25 m × 2 × 6	0.25 m × 3 × 6	0.30 m × 2 × 6	0.30 m × 3 × 6	0.35 m × 2 × 6
Horizontal jump	1.1 m × 2 × 6	1.1 m × 3 × 6	1.2 m × 2 × 6	1.2 m × 2 × 6	1.3 m × 2 × 6
Contacts number	60	90	60	90	60

H, height; S, sets; R, reps.

### Statistical analyses

Statistical analyses were performed using the SPSS 22 program for Windows (SPSS, Inc., Armonk, NY: IBM Corp). The Kolmogorov–Smirnov test was used to verify the normality of all variables (36). Data are presented as mean (SD), and as median values for skewed variables. Initial between-group differences were analyzed using independent *t*-tests, and the effect of the intervention was determined by 2-way analyses of variance [group (PG vs. CG) x time (pre vs. post)]. To evaluate within-group pre-to-post performance changes, paired sample *t*-tests were applied. Percentage changes (delta-change) were calculated as [(post-training value—pre-training value)/pre-training value] * 100. Effect sizes were calculated by converting partial eta squared values to Cohen's *d* [classiﬁed as small (0.00 ≤ *d* ≤ 0.49), medium (0.50 ≤ *d* ≤ 0.79), and large (*d* ≥ 0.80)] (37). Training-related effects were assessed by 2-way analyses of variance (group × time). Statistical significance was set at *p* < 0.05, whether a positive or a negative difference was seen (i.e., a 2-tailed test was adopted). The reliabilities of all dependent variables were assessed by calculating intra-class correlation coefficients (2-way mixed) and coefficients of variation.

## Results

No athlete missed more than 10% of the total training sessions and/or more than two consecutive sessions, so it was not necessary to exclude any participants from the study.

### Reliability of the tests

Test-retest reliabilities were generally above the accepted threshold, with intra-class correlation coefficients ranging from 0.93 to 0.98, and coefficients of variation of 2.1% to 9.2% (Table 2).

**Table 2:** Reliability and variability of change of direction and jump tests.

	ICC	95% CI	CV
T-half	0.974	0.944–0.988	2.1
SJ	0.986	0.971–0.994	8.5
CMJ	0.979	0.955–0.990	7.5
SLJ	0.932	0.852–0.968	9.2

CI, confidence intervals; CV, coefficient of variation; CMJ, counter-movement jump; ICC, intraclass correlation coefficient; SJ, squat jump; T-half, Modified agility *T*-test; SLJ, standing long jump.

### Between-group differences at baseline

There were no signiﬁcant initial intergroup differences for any of the dependent variables.

### Training-related effects

All data, collected after the 10-week intervention, showed significant increases for both PG and CG. With a group × time interaction, the PG enhanced change of direction [i.e., T-half (*p *< 0.001; *d* = 1.95)]; and jump performance [i.e., SJ (*p *= 0.009, *d* = 0.72), CMJ (*p *= 0.005, *d* = 0.79) and SLJ (*p *< 0.001, *d* = 2.25)] compared to the controls (Table 3). Of the same, 3 of 4 repeated sprint ability scores increased significantly in the plyometric relative to the control group [*p *< 0.001, *d* = 1.11 (large); *p *< 0.001, *d* = 1.23; and *p *< 0.001, *d* = 1.21, in RSA-BT, RSA-MT and RSA-TT respectively] (Table 4). Controversially, group × time effects showed no significant difference in both 1-RM bench press and half squat performance between PG and CG (Table 3). Regarding the force velocity performance, the PG enhanced 3 of 4 force velocity scores [*p *< 0.001, *d* = 1 (large); *p *< 0.001, *d* = 1.13; and *p *= 0.012, *d* = 0.72 for PP_UL_ (W), PP_UL_ (W.kg^−1^) and VO_UL_ respectively] for the upper limb performance, and 2 of 4 force velocity scores [*p *= 0.045, *d* = 0.56; and *p *= 0.021, *d* = 0.65 for PP_LL_ (W) and PP_LL_ (W.kg^−1^), respectively] for the lower limb performance. However, F0_UL_, V0_LL_ and F0_LL_ remained unchanged (Table 4).

**Table 3:** Change of direction, jump, repeated sprint ability, and muscular strength test performances in plyometric and control group before and after 10-week intervention.

	Control group (*n* = 14)					Plyometric group (*n* = 14)					Anova group x time interaction	
	Pre	Post	%Δ change	Paired *t* test		Pre	Post	%Δ change	Paired *t* test		*p*	Cohen’s *d*
				*p*	Cohen’s *d*				*p*	Cohen’s *d*		
**Change of direction**												
T-half (s)	7.49 ± 0.16	7.42 ± 0.18	0.8 ± 0.8	0.001	0.43	7.47 ± 0.16	6.70 ± 0.25	10.4 ± 3.1	<0.001	3.81	<0.001	1.95 (large)
**Jump**												
SJ (cm)	22.7 ± 2.3	23.7 ± 1.8	4.7 ± 4.6	0.001	−0.50	22.4 ± 1.6	26.4 ± 1.8	18.3 ± 2.4	<0.001	−2.44	0.003	0.85 (large)
CMJ (cm)	23.9 ± 2.2	24.9 ± 1.9	4.1 ± 3.5	<0.001	−0.50	24.3 ± 1.4	29.3 ± 1.7	20.7 ± 3.1	<0.001	−3.33	<0.001	1.15 (large)
SLJ (m)	1.52 ± 0.15	1.69 ± 0.17	12.2 ± 13	0.003	−1.10	1.50 ± 0.13	1.86 ± 0.15	24.5 ± 13.9	<0.001	−2.66	0.033	0.60 (medium)
**Repeated sprint**												
RSA-BT (s)	7.54 ± 0.07	7.50 ± 0.06	0.5 ± 0.4	<0.001	0.64	7.54 ± 0.07	7.34 ± 0.08	2.6 ± 0.6	<0.001	2.76	<0.001	1.11 (large)
RSA-MT (s)	7.70 ± 0.07	7.66 ± 0.06	0.5 ± 0.5	0.001	0.64	7.71 ± 0.08	7.50 ± 0.08	2.7 ± 0.1	<0.001	2.72	<0.001	1.23 (large)
RSA-TT (s)	46.22 ± 0.42	45.97 ± 0.35	0.5 ± 0.5	0.001	0.67	46.26 ± 0.48	44.99 ± 0.48	2.7 ± 0.1	<0.001	2.75	<0.001	1.21 (large)
RSA-FI (%)	2.16 ± 0.60	2.16 ± 0.55	1.9 ± 19.5	1.000	0.00	2.31 ± 0.71	2.21 ± 0.78	4.9 ± 22.4	0.419	0.14	0.759	0.08 (small)
**1-RM**												
1-RM Bench press (kg)	35.3 ± 10.4	39 ± 10.7	11.2 ± 5.6	<0.001	−0.36	35.9 ± 10.7	42.9 ± 10.3	22.3 ± 17.2	<0.001	−0.71	0.561	0.16 (small)
1-RM Half squat (kg)	73.3 ± 13.4	79.5 ± 14.3	8.6 ± 2.3	<0.001	−0.46	72.2 ± 16	73.7 ± 19.1	6.1 ± 31.3	0.770	−0.09	0.580	0.15 (small)

T-half, Modified agility *T*-test; CMJ, countermovement jump; SLJ, standing long jump; RM, repetition maximal; RSA, repeated sprint ability; BT, best time; MT, mean time; TT, total time; FI, fatigue index; SJ, squat jump.

**Table 4:** Force-velocity test performances in plyometric and control group before and after 10-week intervention.

	Control group (*n* = 14)					Plyometric group (*n* = 14)					Anova group x time interaction	
	Pre	Post	%Δ change	Paired *t* test		Pre	Post	%Δ change	Paired *t* test		*p*	Cohen’s *d*
				*p*	Cohen’s *d*				*p*	Cohen’s *d*		
**Upper limb**												
PP (W)	146 ± 24	146.5 ± 10.7	2.7 ± 17.6	0.948	−0.03	144.8 ± 25.9	186 ± 20.5	30.8 ± 17.3	<0.001	−1.83	0.001	1.00 (large)
PP (W.kg^−1^)	1.9 ± 0.2	1.9 ± 0.2	3.8 ± 18.7	0.688	0.00	1.9 ± 0.3	2.4 ± 0.2	31.5 ± 16.6	<0.001	−2.04	<0.001	1.13 (large)
V0 (rpm)	87.3 ± 17.3	84.2 ± 8.6	0.1 ± 21.5	0.525	0.24	88.8 ± 17.1	106.5 ± 14.8	21.9 ± 14.6	<0.001	−1.15	0.012	0.72 (medium)
F0 (N)	6.5 ± 1.2	7.1 ± 0.7	11.6 ± 23.9	0.108	−0.63	6.6 ± 0.9	7.3 ± 0.7	11.1 ± 12.3	0.005	−1.42	0.816	0.06 (small)
**Lower limb**												
PP (W)	337.8 ± 37.9	355.4 ± 35.9	5.3 ± 3	<0.001	−0.49	345.9 ± 48.5	407.1 ± 35.9	18.5 ± 8	<0.001	−1.49	0.045	0.56 (medium)
PP (W.kg^−1^)	5.3 ± 0.5	5.4 ± 0.5	3.3 ± 3.3	0.003	−0.21	5.5 ± 0.8	6.5 ± 0.7	11.5 ± 7.1	<0.001	−1.38	0.021	0.65 (medium)
V0 (rpm)	162.9 ± 18.8	164.1 ± 22.4	0.8 ± 8.8	0.767	−0.06	164.8 ± 21.3	163.9 ± 26.6	0.6 ± 8.9	0.831	0.04	0.866	0.06 (small)
F0 (N)	7.8 ± 0.5	8.7 ± 1.1	11.1 ± 12.8	0.006	−1.09	7.8 ± 0.5	9.3 ± 1.2	20.3 ± 15.3	<0.001	−1.69	0.146	0.40 (small)

PP, peak power; V0, maximal pedaling velocity; F0, maximal braking force.

## Discussion

The current study aimed the effectiveness of a 10-week PT intervention in improving change of direction, jumping ability, repeated sprint ability, and muscular strength and power in youth female handball players. With the exception of muscular strength (1-RM bench press and 1-RM half squat), performance on these selected measures was significantly enhanced by PT in comparison with the standard regimen.

Change-of-direction capacity refers to a movement where no immediate reaction to a stimulus is required, so the direction change is preplanned (19). It has been proved among the key qualities in a handball match (38), and is affected by strength, power, and speed (19). The present finding revealed a significant improvement in COD (i.e., T-half) in the PG compared to the CG. In the literature, several studies examined the impact of plyometrics and found increases (11, 13) and decreases (22, 39) in COD performance. Discrepancy between studies may be explained by numerous factors (i.e., training level, gender, age, sport activity, or familiarity with plyometrics) and training variables (i.e., surface and type of PT, rest period between sets and training sessions, and the principle of specificity). The possible mechanisms of COD improvements could be the result of force gain and high-power output and the ability to efficiently use the stretch-shortening cycle in ballistic movements (11, 13). In fact, PT enhances the neuromuscular system's ability to generate and control force rapidly. This type of training stimulates the stretch-shortening cycle, which involves rapid muscle lengthening followed by a forceful contraction (18). The neuromuscular system becomes more efficient in coordinating the timing and recruitment of muscle fibers, leading to improved power production during movements involved in COD tasks (18). The change-of-direction tasks are amongst the most frequently performed activities during matches, and it is an important physical fitness attributes in handball (40). For it, coaches must include PT exercises combined with a change of direction exercise. In future studies, it is possible to include PT exercises combined with reactive agility exercise (nonplanned change of direction).

The present results demonstrated significant improvement in all jumps performances in the PG relative to CG. Some studies reported increases in vertical and horizontal jump after PT in young female athletes (11, 13). Similar to our training program, Hammami et al. (13) found increases in Squat and CMJ and horizontal jump performance in young female handball players. The PT effects on vertical jumping performance in female athletes were reported in a published meta-analysis (20), which demonstrated that less than 10 weeks of plyometrics generated small CMJ performance improvements (ES = 0.58) in female athletes (20). The efficiency of the stretch-shortening cycle, neural drive to the agonist muscles, muscle activation strategies like intermuscular and intramuscular coordination, changes to muscle size and architecture, and changes to single-fiber mechanics are just a few of the neuromuscular-related adaptations that may interact to improve jump performance (18, 20, 23). Given the substantial empirical evidence demonstrating the effectiveness of this method of training, it is not surprising that improvements in jumping performance were caused by the plyometric protocol (18, 20, 23). According to existing research, the eccentric phase of a plyometric exercise with a ground contact time of less than 250 ms demonstrates the longest stretch-shortening cycle stimulation, which maximizes performance (41). The primary neuromuscular mechanisms behind training-induced performance increases must still be investigated in more detail in new research.

Findings of this study indicated that plyometrics combined with traditional handball training induced large significant improvement in RSA scores (best, mean and total time), but no significant change in fatigue index. The lack of signiﬁcant change in fatigue index could be due to the poor reproducibility of this selected measure (30). This was in accordance with previous studies that also showed trivial to moderate effect sizes for best time, total time, and fatigue index (11, 17). PT effects on the final results in progressive load tests could be explicated by reduced contact time with the surface, improved tendon and muscle rigidity, increased mechanical output caused by the muscles and tendons’ elastic attributes, and better movement economy as a whole. After plyometric training, improvement in RSA scores due to higher number of recruited motor-unit and better motor-unit synchronization, increasing firing frequencies, better stretch-shortening cycle efficiency, or increased musculotendinous stiffness (18, 31).

Compared with the performance improvements seen in the change of direction, jumping and RSA tests, there were no observable enhancement in both 1-RM bench press and half-squat PT in our study. This could have occurred due to the multidimensional demands of handball training, but with no appreciable improvement in the 1-RM bench press and 1-RM half-squat performance of the intervention groups, it is unlikely that the plyometrics, as delivered in the current program, exerted any effect on performance in the 1-RM tests. That the plyometrics seemed to exert a preferential impact on SJ and CMJ is unsurprising, given the similarity of the training stimulus to the respective tests used. Likewise, the specificity of plyometric training, which does not contain exercises based on additional load (i.e., moving a load). Although, plyometric exercises primarily target the stretch-shortening cycle and focus on generating power and explosiveness. On the other hand, maximal strength exercises like the half-squat and bench press primarily aim to increase maximum force production. The specific adaptations required for each type of training may differ, and improvements in one may not directly translate to improvements in the other. For instance, Vissing et al. (42) have previously reported the sensitivity of certain physical attributes to training stimuli that share similar characteristics. They demonstrated that a greater extent increased SJ and CMJ by plyometrics than it was by conventional resistance training, thus reinforcing the principle of training specificity (43). To the authors’ knowledge, only a few studies have previously focused on the effects of PT on 1-RM bench press or half squat performance in young female athletes (44, 45). The authors noted that 12-week PT can enhance strength (i.e., back squat performance) in female adolescent handball players aged 14.9 years old (45). The disagreement from present findings could be explained by methodological differences (duration of program; the type of exercise; the instrument used: dynamometer test or 1-RM test).

Power is a paramount performance determinant in handball (7). The results of our study showed moderate to large improvements for both upper and lower limb force-velocity performances (Table 4). To the authors’ knowledge, no study has previously addressed the effects of PT on force-velocity performance in young female athletes. Using similar PT on male players, Chelly et al. (16) reported increases in upper limb force velocity scores (absolute peak power: 27.4%) and peak relative to body mass (28.7%) following an 8-week bi-weekly course of upper limb plyometric training in junior male handball players. Similarly, Chelly et al. (34) found increases of absolute Peak power and peak power relative to body mass. However, no increases of peak power per unit of muscle volume or thigh muscle volume was shown after 8-week PT in male soccer players aged 19 years. Conversely, Hammami et al. (12) failed to find any significant change in all force-velocity scores after 8-week plyometric training in male soccer players (age = 15.8 years). Regarding V0 parameter, our data revealed increases in V0 upper limb, nevertheless V0 lower limb remained unchanged. According to our findings, Chelly et al. (16) demonstrated increases in V0 upper limb performance. However, for both upper and lower limb the F0 score remained unchanged. This coincides with the results of the literature (16, 17, 34). Discrepant findings probably reflect differences in methodology (for instance, the testing of post-adolescent vs. much younger players; elite or professional players vs. regional level players; the format of the plyometric exercises, the frequency, duration and progression of training, and its timing relative to the playing season). In terms of training intensity, volume, and exercise selection we followed the principle of progressive overload, starting with lower intensities, single-joint exercises, and less complex exercise techniques, and progressing to higher intensities, multi-joint exercise, and more complex techniques. In brief, the present study outcomes showed that either plyometric training is equally effective training interventions in improving young female handball players’ force-velocity performance.

This study has certain limitations that should be taken into consideration. Firstly, only physical performance was evaluated. Physiological data may provide some neuromuscular mechanisms responsible for the observed findings. Secondly, we did not assess other anthropometric measurements such as limb muscle volume, thigh muscle volume, cross-sectional area, and peak power per unit for both upper and lower limb, which would allow us to make assumptions. Thirdly, although the players were questioned whether they had a typical menstrual cycle, or if they used hormonal contraception, it was not possible to align their training according to their cycles due to the group training. As it was impossible that their cycles ran in tandem with each other, this was not taken into account.

## Conclusion

This study demonstrated that a short-term, in-season PT program in place of some handball-specific drills are undoubtedly able to enhance physical fitness measures (i.e., change of direction, jumping, RSA, strength, and power) in youth female handball players. These outcomes could help coaches and practitioners to better structure their training programs concerning the types of training used. PT is a time-efficient and highly helpful method for improvement of both upper and lower limbs physical performance in youth female handball players. Supplementary studies are needed to investigate the effects of PT on muscle morphology and neural adaptations. Similarly, it will be interesting to explore the impact of maturation status as a potential moderator variable.

## Data Availability

The raw data supporting the conclusions of this article will be made available by the authors, without undue reservation.
